# Multi-task deep learning models for mechanism-based prediction of developmental and reproductive toxicity (DART) using ToxCast bioassays

**DOI:** 10.3389/ftox.2026.1751644

**Published:** 2026-02-04

**Authors:** Siyeol Ahn, Hojun Jung, Jinwon Hwang, Donghyeon Kim, Hyunjun Kim, Wooseok Kim, Yunjung Lee, Changwon Lim, Jinhee Choi

**Affiliations:** 1 School of Environmental Engineering, University of Seoul, Seoul, Republic of Korea; 2 Department of Statistics and Data Science, Chung-Ang University, Seoul, Republic of Korea; 3 Department of Smart Cities, Chung-Ang University, Seoul, Republic of Korea

**Keywords:** ToxCast, toxicity prediction, deep learning, developmental and reproductive toxicity, new approach methodologies

## Abstract

Developmental and reproductive toxicity (DART) testing has traditionally relied on animal studies, which are costly, time-consuming, and ethically constrained. To advance new approach methodologies (NAMs), we developed a mechanism-informed deep learning framework for predicting DART using *in vitro* bioactivity data from 23 ToxCast assays mechanistically linked to key developmental and reproductive pathways. Four state-of-the-art (SOTA) deep learning architectures (DGCL, TransFoxMol, MolPath, and MolFormer) were evaluated to address performance limitations commonly observed in traditional supervised learning approaches. Each model was fine-tuned using the curated ToxCast dataset, with the F1 score serving as the primary evaluation metric. Among these, the DGCL model consistently outperformed baseline machine learning algorithms, including random forest, XGB, GBT, decision tree, and logistic regression. Extending DGCL to a multi-task learning framework further improved model stability and performance for endpoints with limited active data. External validation with 91 reference chemicals curated and verified by the ECVAM ReProTect program demonstrated balanced predictive performance (F1 = 0.68), confirming the reliability and generalizability of the fine-tuned DGCL model. By leveraging advanced deep learning architectures, the model effectively handles mechanistically diverse and imbalanced assay data with limited active samples, resulting in improved predictive performance across DART-related effects. Overall, this study demonstrates the potential of integrating mechanistic bioassay information with deep learning to develop reliable, mechanism-based, and non-animal methods for DART prediction and potential regulatory application.

## 1 Introduction

Developmental and reproductive toxicity (DART) represents a highly complex and resource-demanding area within chemical safety assessment (Martin et al., 2009). Conventional DART testing, such as those described in the OECD Test Guidelines 414, 421, 422, and 443, relies heavily on animal models to provide apical endpoint data relevant to human health (OECD, 2018; OECD, 2025). While indispensable for hazard identification, these *in vivo* studies are costly, time-consuming, and ethically constrained. Moreover, species-specific differences often limit their translational relevance to humans, emphasizing the need for more efficient and mechanistically informed alternatives (Krewski et al., 2010; Vargesson, 2015). These limitations underscore the need for mechanistically informed and human-relevant new approach methodologies (NAMs) that can complement conventional animal-based testing and support early-stage screening and prioritization of chemicals for DART-related effects.

NAMs, including *in silico*, in chemico, and *in vitro* systems, are increasingly recognized as important tools for mechanistic toxicity evaluation (Parish et al., 2020). Among these, high throughput screening programs such as the U.S. EPA ToxCast have generated extensive bioactivity data across diverse molecular targets and biological pathways (Judson et al., 2010; Jeong et al., 2022). These datasets provide a valuable basis for identifying assays relevant to DART mechanisms (Sipes et al., 2011; Abedini et al., 2021). Recent studies have also emphasized integrating human biology information to define DART related biomarkers and pathway level associations, thereby improving the mechanistic interpretability of *in vitro* data and supporting their use in predictive toxicology (Kim et al., 2025a).

With the rapid advancement of artificial intelligence (AI), data driven modeling has become a key approach in predictive toxicology (Jeong and Choi, 2022). Recent advances have enabled the application of machine learning techniques to systematically analyze large scale *in vitro* bioactivity data and uncover relationships between chemical structures and biological responses (Huang et al., 2016; Kim and Choi, 2025). While these models have demonstrated the feasibility of mechanism-based toxicity prediction, their performance is often limited by endpoint specific architectures, data imbalance between active and inactive chemicals, and insufficient integration across related biological pathways (Seal et al., 2025).

Recent advances in state-of-the-art (SOTA) artificial intelligence methods, particularly graph based deep learning and multi-task learning, offer new opportunities to address these limitations. Graph-based models can represent chemical structures as relational graphs, allowing the extraction of intricate substructural features relevant to biological activity (Hong and Kwon, 2025). Meanwhile, multi-task learning can enhance model robustness by exploiting shared representations among correlated toxicity endpoints (Lin et al., 2024). Despite their demonstrated success in pharmacology and bioactivity prediction, their systematic application to DART remains limited.

In our previous study (Kim and Choi, 2025), we curated 23 ToxCast bioassays mechanistically associated with DART and developed traditional machine learning models to predict developmental and reproductive effects. Although these models yielded reasonable results, their overall accuracy remained limited, highlighting the need for improved modeling approaches. Accordingly, enhancing predictive performance became a primary objective of the present study. To address this limitation, we developed an advanced, mechanism-informed deep learning framework using the same 23 DART-related ToxCast bioassays mapped to key molecular pathways. We systematically compared the predictive performance of deep learning and machine learning algorithms and evaluated the added contribution of a multi-task learning setup based on shared representation learning for improving model stability. In addition, we conducted external validation using reference chemicals from the ECVAM ReProTect program to evaluate generalizability and robustness (Pazos et al., 2010). Through this approach, we aimed to establish a more accurate and reliable mechanism-based model for DART prediction.

## 2 Methods

### 2.1 Study design

**FIGURE 1:**
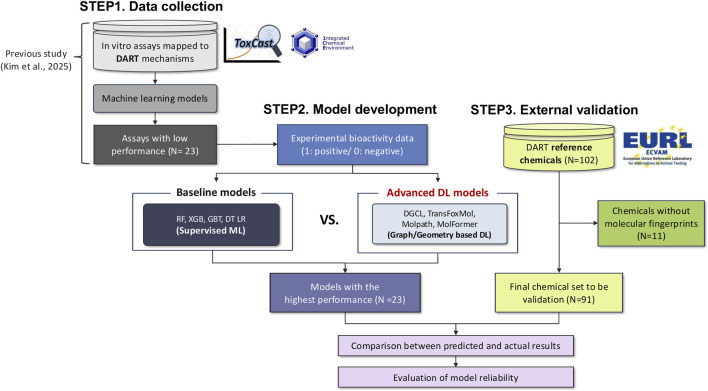
Workflow of study.

This study was organized into three major steps, which included data collection, model development, and external validation (Figure 1). In the first step, we used 23 ToxCast bioassays associated with DART that had been selected in a previous study based on mechanistic relevance and statistical correlation with *in vivo* reference data (Kim et al., 2025a). These assays showed limited predictive performance when conventional machine learning approaches were applied, which prompted the present effort to enhance assay-level performance using more advanced deep learning methods.

Next, we generated multiple molecular representations, including molecular fingerprints, molecular graphs, and three-dimensional structural features, and used them to develop five machine learning (ML) and four deep learning (DL) models for each assay. The best-performing ML and DL models for all 23 assays were identified based on F1-scores, and their predictive performances were compared. To examine the effect of task configuration, the DL models were also trained in both single-task and multi-task settings to assess whether shared learning improved performance.

In the final step, the optimized multi-task deep learning model was evaluated using 91 reference chemicals from the EURL-ECVAM ReProTect program to assess predictive reliability and generalizability.

### 2.2 *In vitro* data collection from ToxCast and annotation of DART-related modes of action

ToxCast data were obtained from the U.S. EPA’s publicly available summary file in the invitroDB v4.1 dataset (data release: 14 September 2023) (https://clowder.edap-cluster.com/spaces/647f710ee4b08a6b394e426b). In this study, we used 23 ToxCast bioassays for model development. These assays were selected in a previous study based on their mechanistic relevance to developmental and reproductive toxicity (DART) and their showed associations with *in vivo* DART outcomes. (Supplementary Table S1) (Kim et al., 2025a). For each assay, hit call data were applied, where bioassay outcomes were labeled as positive (1) for confirmed active class and negative (0) for inactive class (Filer et al., 2017).

Mechanistic annotations used to group the selected 23 assays were obtained from the NTP Integrated Chemical Environment (ICE) database (query date: 03 January 2025) (https://ice.ntp.niehs.nih.gov/DATASETDESCRIPTION?section=cHTS). Among the assays available in ICE, 201 were mapped to DART related mechanisms curated by NICEATM and ICCVAM (Abedini et al., 2021). These mechanistic annotations, which are based on the biological processes described by Kleinstreuer et al. (2011) and van gelder et al. (2010), were used to assign modes of action associated with DART-related endpoints and to classify the selected ToxCast assays into mechanistic categories.

### 2.3 Data preprocessing and balancing

Following data collection, a series of preprocessing procedures were applied to the ToxCast assay dataset. The simplified molecular input line entry system (SMILES) identifiers of chemicals included in the assays were obtained from the U.S. EPA CompTox Chemicals Dashboard (https://comptox.epa.gov/dashboard).

SMILES canonicalization was performed using RDKit to standardize molecular representations and ensure consistent handling of aromaticity and resonance-related structures. As an additional quality-control measure, we removed compounds containing metals and any residual entries annotated as salts or ionic mixtures, since such structures may lead to inconsistent valence handling and unstable graph construction in standard cheminformatics pipelines.

For the remaining compounds, SMILES strings were processed with RDKit to construct molecular objects and converted into 2D molecular graph representations. Throughout this process, kekulization was not applied and RDKit aromaticity perception was preserved, so that aromatic bonds and atom flags were retained consistently across all deep learning models. This approach reduces potential ambiguity in aromatic systems and supports uniform encoding of aromatic rings in downstream graph encoders. For geometry-aware models, additional geometry-dependent inputs derived from 3D coordinates were incorporated. Deep learning model-specific inputs were then generated as described in Section 2.5. In addition to graph-based representations used for deep learning models, molecular fingerprints were generated for baseline machine learning models using the RDKit library (version 2023.9.6). Four types of fingerprints were employed, including Morgan, MACCS, RDKit topological, and Layered fingerprints. (Morgan, 1965; Durant et al., 2002; Riniker and Landrum, 2013). Morgan fingerprints (ECFP4) were generated with a radius of 2 and encoded as 1,024-bit binary vectors. RDKit topological fingerprints were constructed by hashing atom–bond paths of length 1–7 into a 2,048-bit vector. MACCS fingerprints consisted of 166 predefined structural keys, resulting in a 166-bit representation. Layered fingerprints were encoded as 2,048-bit vectors designed to capture layered atom environments and hierarchical structural features.

ToxCast hitcall data exhibited substantial class imbalance, with inactive outcomes predominating overactive ones across most assays. In our previous work, we evaluated multiple resampling strategies to assess their influence on model performance in toxicity prediction (Bae et al., 2021). Based on those findings, the synthetic minority oversampling technique (SMOTE) was adopted to augment the representation of the minority (active) class in the present dataset (Chawla et al., 2002). Data balancing was implemented using the imbalanced-learn Python library (version 0.9.1).

### 2.4 Data splitting

To ensure a fair comparison between machine learning and deep learning models, we created three independent dataset splits using different random seeds (Seed 1, Seed 2, and Seed 3). For each seed, the dataset was randomly divided into training (80%), validation (10%), and test (10%) subsets. This multi-seed strategy minimized random variation during splitting and allowed us to assess model robustness across different random partitions. Each model was trained and evaluated on all three seed-based splits, and final performance metrics were reported as the mean and standard deviation of results across seeds. In addition, a randomized-label (y-shuffle) baseline analysis was performed to verify that performance exceeded chance. For each fixed seed-based split, we randomly permuted the training labels 100 times while keeping the split membership unchanged and reported the resulting null distribution together with an AUC-based separation metric in Supplementary Table S2.

### 2.5 Molecular representation learning in deep learning model

To develop predictive deep learning models for DART mechanisms, it is essential to convert complex chemical structures into informative, machine-readable formats. Molecular representation learning plays a pivotal role by enabling the transformation of chemical structures into numerical representations. The objective is to derive embeddings that retain molecular topology, substructural features, and spatial configurations, suitable for deep learning. This process can be broadly categorized into three paradigms: sequence-based, graph-based, and geometry-based representations (Yu et al., 2021). Detailed model architecture and mathematical formulations are provided in Supplementary Information S1.

#### 2.5.1 Sequence-based representation

Sequence-based representations express molecules as symbolic strings, such as SMILES, that describe atomic connectivity. This enables the reuse of natural language processing architectures and self-supervised objectives, including masked language modeling and sequence reconstruction, to learn chemical patterns from large datasets of molecular strings (Honda et al., 2019; Chithrananda et al., 2020). However, these approaches often fail to capture spatial, topological and geometric information that define molecular geometry structures (Guo et al., 2022).

#### 2.5.2 Graph-based representation

Graph-based representations describe molecules as a 2D graph $G=\left(V,E\right)$ , where atoms correspond to nodes V and bonds to edges. Node features typically encode atomic identity and local chemical environment, while edge features encode bond type and aromaticity information. Graph Neural Networks (GNN) transform this molecular graph into a compact embedding through local aggregation of information across the graph structure (Gilmer et al., 2017). A unifying perspective on many GNNs is the Message Passing Neural Network (MPNN) (Scarselli et al., 2009). It propagates information between nodes through iterative message passing to derive contextual atom features. Popular architectures including Graph Convolutional Networks (GCN), Graph Isomorphism Networks (GIN), and Graph Attention Networks (GAT) can be viewed as specific instances of this general message-passing scheme, primarily distinguished by their choice of aggregation and weighting functions (Kipf and Welling, 2016; Veličković et al., 2017; Xu et al., 2018). The model utilized in this study, TransFoxMol, DGCL, and MolPath, extend this paradigm in complementary ways. For all graph-based models, atom-level representations were aggregated into a fixed length molecule-level embedding using readout and pooling strategy implemented in each model’s official repository. To ensure reproducibility, all three models were trained on the unified 2D molecular graphs generated under the consistent preprocessing and graph construction methods described in Section 2.3. TransFoxMol is a supervised, transformer based multi-modal model, which learns molecular representations from 2D graphs by extracting local structure features with GNN and modeling global features using the transformer’s self-attention mechanism, thereby capturing both short-range chemical environments and long-range dependencies within a unified architecture (Gao et al., 2023). DGCL is a self-supervised learning framework based on contrastive learning. During pretraining, it learns robust molecular embeddings by encoding the same molecular graph through two different GNN encoders (GIN and GAT) and optimizing a contrastive objective that aligns the encoder-derived representations for the same molecule while separating representations across different molecules within a batch (Jiang et al., 2024). MolPath is a supervised, chain aware GNN designed to capture the long-range dependencies that are common in molecular structures. It enhances representation learning by incorporating path convolution and initial residual difference connection (IRDC) module, which facilitates effective propagation of information across distant substructures in the molecular graph. (Wang et al., 2024).

#### 2.5.3 Geometry-based representations

Geometry-based representations incorporate 3D spatial information such as atomic coordinates, interatomic distances, and bond angles. The effectiveness of geometric deep learning models in molecular prediction tasks is demonstrated by architectures including ChemRL-GEM (Fang et al., 2022). Molformer model evaluated in this study is a supervised, 3D geometric transformer-based model that explicitly incorporates 3D molecular structure (Wu et al., 2023). It operates on heterogeneous molecular graphs (HMG), which uniquely consist of both atom-level and motif-level nodes. To effectively model interactions between multi-level nodes, Molformer utilizes its heterogeneous self-attention (HSA) mechanism. To ensure reproducibility, Molformer was trained using the same preprocessed molecular inputs described in Section 2.5.0, while incorporating 3D geometry-dependent inputs derived from 3D coordinates.

### 2.6 Model adaptation and hyperparameter optimization

**FIGURE 2:**
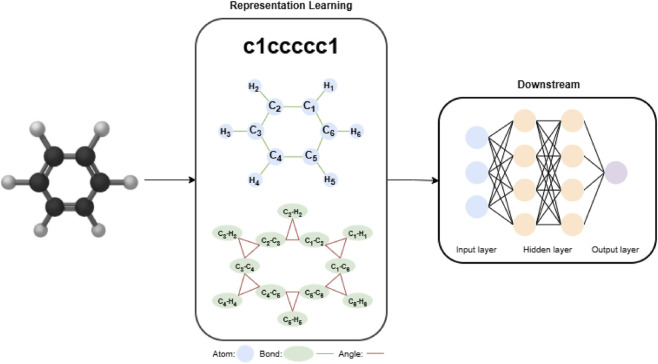
End-to-end workflow of the molecular representation learning and prediction framework based on deep learning.

For downstream predictive modeling, all four architectures used the molecule-level embeddings produced by their respective encoders as inputs to a shared multilayer perceptron (MLP) classification head. The overall workflow for molecular property prediction is illustrated in Figure 2. The structure of the MLP classifier was kept consistent across all models, and the complete set of tuned hyperparameters is summarized in Supplementary Table S3. During downstream adaptation, each model was trained following the experimental procedures and implementation recommendations described in its original paper, ensuring that the behavior of each architecture remained faithful to its intended design. Across all experiments, model selection was based on validation performance, and early stopping was employed to prevent overfitting.

For TransFoxMol, the model was trained from scratch without external pretraining, and Bayesian optimization was used during hyperparameter search to identify the best-performing configuration. For DGCL, the contrastively pretrained GIN and GAT encoders were used as fixed feature extractors. Their graph-level embeddings, concatenated with molecular fingerprints, were passed to the shared classifier, while encoder parameters remained frozen. For MolPath, the pretrained path- and chain-aware encoder was fully fine-tuned on ToxCast assay data, allowing the model to adapt its shortest-path and long-range dependency modeling to the endpoints. For Molformer, the pretrained heterogeneous Transformer encoder and AFPS readout were transferred to the ToxCast assay data. Depending on the configuration, the encoder was either fully fine-tuned or partially frozen during training. Training and validation loss curves for the DGCL model, illustrating model convergence and early stopping behavior, are provided in the Supplementary Figure S1.

### 2.7 Implementation of multi-task learning approach

In predictive modeling, two fundamental learning paradigms are commonly used to address multi-dimensional problems, with approaches typically categorized into single-task learning and multi-task learning.

STL trains separate models, each dedicated to a single prediction target. In this study, an independent model was trained for each of the 23 ToxCast bioassays, resulting in 23 models per architecture. Although this design enables task-specific optimization, it limits the model’s ability to exploit potential correlations among biologically related assays.

In contrast, multi-task learning aims to learn multiple prediction tasks simultaneously using a single unified model. In the multi-task learning setting, one model was trained across all 23 assays, enabling shared representations across tasks. Such shared representation learning allows the model to capture underlying biological relationships among assays. By evaluating both single-task learning and multi-task learning configurations, this study systematically compares task-specific and task-shared learning strategies to determine whether shared representations across biologically related assays improve molecular property prediction performance.

### 2.8 Training baseline algorithms

Five machine learning algorithms were employed in this study, including Logistic Regression (Logistic), Decision Tree (DT), Random Forest (RF), Gradient Boosting (GBT), and Extreme Gradient Boosting (XGB). For each algorithm, four different molecular fingerprint representations (Morgan, MACCS, RDKit, Layered) and approximately one hundred hyperparameter combinations were evaluated using a grid search approach. The specific hyperparameters considered for each algorithm are summarized in Supplementary Table S3. For conventional statistical models, model complexity was primarily controlled through regularization using penalty terms, whereas for tree-based ensemble models, major parameters included the number of trees and their maximum depth. Model training was conducted using the scikit-learn package (version 1.5.1). The detailed development procedure baseline model development was described in our previous study (Kim et al., 2025b).

### 2.9 Performance evaluation

Model performance was comprehensively assessed using general performance metrics, including accuracy, precision, recall, AUC, and F1 score (Huang and Ling, 2005). The selection of performance metrics can vary depending on the study objective and experimental design. While accuracy is a widely used indicator and suitable for evaluating models under balanced conditions, it is less informative when the dataset is imbalanced. In line with our previous findings, the F1 score was identified as the most reliable indicator for toxicity prediction tasks (Kim et al., 2024a; Kim et al., 2024b). Therefore, the F1 score was used as the primary criterion to identify the best performing algorithm among the five machine learning models and the best performing architecture among the four deep learning models developed for each assay.

### 2.10 External validation dataset from ECVAM ReProTect

For external validation, we utilized the reference chemical set curated and validated by the European Centre for the Validation of Alternative Methods (ECVAM) of the Joint Research Centre (JRC) under the ReProTect program (Pazos et al., 2010). The original dataset consists of 102 chemicals; however, 11 chemicals without valid molecular representations were excluded, resulting in a final set of 91 chemicals used for validation. These chemicals were assessed for three target effects (developmental toxicity, male fertility, and female fertility), and their positive or negative labels were assigned based on integrated evaluations of available *in vivo* evidence, as classified within the ReProTect program. The dataset contained 75 positive and 16 negative chemicals (Table 1). Descriptions of the effects associated with each chemical, along with supporting references, were summarized in the original ReProTect publications, and the complete list of chemicals used for external validation in this study is provided in Supplementary Table S4. This curated dataset was employed to assess the generalizability and predictive reliability of the deep learning models trained on ToxCast bioassay data.

**TABLE 1:** DART evaluation set for binary categorical models. The individual chemical classifications are provided in Supplementary Table S4.

Effect	Positive	Negative	Total
Developmental toxicity	21	6	27
Male fertility	27	5	32
Female fertility	27	5	32
Total	75	16	91

### 2.11 Evaluation of applicability domain

The applicability domain (AD) provides a quantitative criterion for assessing the reliability of model predictions (Weaver and Gleeson, 2008), and only compounds falling within this domain are regarded as yielding reliable predictions. This study used a similarity-based AD analysis approach was adopted, following previously established methodologies (Gou et al., 2022; Kim et al., 2024a). For every query molecule, the Euclidean distance was calculated using MACCS fingerprints to its k = 3 nearest neighbors in the training set. An AD threshold, $D_{T}$ was then defined as, $D_{T}=\bar{y}+Z_{\sigma }$ , represents the mean of the k-nearest-neighbor distances across all training compounds, σ represents the corresponding standard deviation, and the scaling factor Z was set at 0.5. A prediction was considered unreliable and outside the applicability domain if the distance between a test compound and any of its nearest training neighbors exceeded the defined threshold.

## 3 Results and discussion

### 3.1 Data distribution of the DART-related ToxCast assay dataset

To develop predictive models for developmental and reproductive toxicity (DART) mechanisms, 23 ToxCast bioassays were selected in our previous study (Kim et al., 2025a). These assays are mapped to key molecular pathways associated with DART and show statistically significant correlations with *in vivo* DART reference data.

The selected assays include multiple biological mechanisms relevant to DART (Table 2). Steroid hormone–related pathways (n = 9) were the most frequently represented, including estrogen receptor modulation, progesterone receptor modulation, and estrogen metabolic processes, followed by extracellular matrix organization (n = 4) and angiogenic processes (n = 4).

**TABLE 2:** Summary of ToxCast bioassays associated with mechanisms of DART. The detailed assay information is provided in Supplementary Table S1.

Mode of action (NTP ICE)	AEID	ToxCast assay name	Intended target gene	No. of chemicals	Active chemicals (%)
Angiogenic process	174	BSK_4H_MCP1	CCL2	1705	407 (23.87%)
	196	BSK_BE3C_PAI1	SERPINE1	1705	306 (17.95%)
	250	BSK_hDFCGF_PAI1	SERPINE1	1705	430 (25.22%)
	258	BSK_hDFCGF_VCAM1	VCAM1	1705	451 (26.45%)
Aryl hydrocarbon receptor modulation	63	ATG_Ahr_CIS	AHR	4,039	692 (17.13%)
Estrogen metabolic process	907	CEETOX_H295R_ESTRADIOL	ESR1	576	74 (12.85%)
	909	CEETOX_H295R_ESTRONE	ESR1	576	90 (15.63%)
Estrogen receptor modulation	75	ATG_ERE_CIS	ESR1	4,039	848 (21%)
	786	TOX21_ERa_BLA_Antagonist_ratio	ESR1	8,305	1,053 (12.68%)
	2,119	TOX21_ERb_BLA_Antagonist_ratio	ESR2	7,871	1,459 (18.54%)
	2,211	TOX21_ERa_LUC_VM7_Agonist_10 nM_ICI182780	ESR1	7,871	159 (2.02%)
Estrogen-related receptor modulation	2070	TOX21_PGC_ERR_Antagonist	ESRRA	7,871	979 (12.44%)
Extracellular matrix	166	BSK_3C_uPAR	PLAUR	1705	443 (25.98%)
	232	BSK_CASM3C_uPAR	PLAUR	1705	266 (15.6%)
	256	BSK_hDFCGF_TIMP1	TIMP1	1705	300 (17.6%)
	274	BSK_KF3CT_TIMP2	TIMP2	1705	339 (19.88%)
Other developmental signaling transcription factors	66	ATG_BRE_CIS	SMAD1	4,039	365 (9.04%)
Oxidative stress	82	ATG_HIF1a_CIS	HIF1A	4,039	400 (9.9%)
p53 signaling pathway	1,317	TOX21_p53_BLA_p2_ratio	TP53	8,305	828 (9.97%)
Progesterone metabolic process	895	CEETOX_H295R_OHPROG	PGR	576	243 (42.19%)
	913	CEETOX_H295R_PROG	PGR	576	224 (38.89%)
Progesterone receptor modulation	2,127	TOX21_PR_BLA_Antagonist_ratio	PGR	7,871	2008 (25.51%)
Retinoic acid receptor modulation	71	ATG_DR5_RAR_CIS	RARA	4,039	309 (7.65%)

Across these assays, the number of tested chemicals ranged from fewer than 100 to over 7,000, with approximately 7%–26% identified as active. However, the proportion of active chemicals varied considerably across assays. For example, TOX21_ERa_LUC_VM7_Agonist_10 nM_ICI182780 showed only about 2% actives, whereas CEETOX_H295R_ OHPROG exhibited 42%. This variability in activity distribution highlights the intrinsic imbalance across assay endpoints.

### 3.2 Development of models for DART-related mechanistic bioactivity prediction

#### 3.2.1 Performance comparison between deep learning and machine learning models

As a baseline, we first trained five machine learning (ML) algorithms, including Logistic Regression (Logistic), Decision Tree (DT), Random Forest (RF), Gradient Boosting (GBT), and Extreme Gradient Boosting (XGB), using the selected ToxCast DART-related bioassay dataset. Across all tested bioassays, these ML models showed modest predictive performance, with F1-scores ranging from 0.26 to 0.49.

To improve predictive performance, we next developed four deep learning (DL) architectures, including DGCL, TransFoxMol, MolPath, and MolFormer, and systematically compared them with the ML baselines. For each assay, the best-performing model within the ML and DL groups was identified based on the highest F1-score to enable a consistent comparison (Table 3).

**TABLE 3:** Comparison of the best-performing machine learning (ML) and deep learning (DL) models across ToxCast bioassays related to DART. Performances of all tested models are provided in the Supplementary Table S5.

Mode of action	ToxCast assay name	Algorithms		F1 score		ROC-AUC		Precision		Recall	
		ML	DL	ML	DL	ML	DL	ML	DL	ML	DL
Angiogenic process	BSK_4H_MCP1	LR	DGCL	0.4	**0.47**	0.62	**0.71**	0.38	**0.38**	0.43	**0.63**
	BSK_BE3C_PAI1	DT	DGCL	0.36	**0.43**	0.59	**0.72**	0.32	**0.32**	0.44	**0.67**
	BSK_hDFCGF_PAI1	LR	DGCL	0.44	**0.52**	0.61	**0.72**	0.35	**0.4**	0.62	**0.76**
	BSK_hDFCGF_VCAM1	LR	TransFoxMol	0.41	**0.47**	0.61	**0.71**	0.34	**0.46**	0.54	**0.52**
Aryl hydrocarbon receptor modulation	ATG_Ahr_CIS	LR	DGCL	0.36	**0.39**	0.61	**0.68**	0.27	**0.29**	0.54	**0.62**
Estrogen metabolic process	CEETOX_H295R_ESTRADIOL	GBT	DGCL	**0.26**	0.24	0.52	**0.62**	**0.3**	0.19	0.23	**0.34**
	CEETOX_H295R_ESTRONE	DT	DGCL	0.37	**0.38**	0.56	**0.64**	**0.3**	0.28	0.61	**0.64**
Estrogen receptor modulation	ATG_ERE_CIS	LR	DGCL	0.48	**0.49**	0.67	**0.71**	0.39	**0.42**	**0.65**	0.59
	TOX21_ERa_BLA_Antagonist_ratio	LR	DGCL	0.31	**0.44**	0.63	**0.78**	0.28	**0.33**	0.36	**0.69**
	TOX21_ERb_BLA_Antagonist_ratio	RF	DGCL	0.48	**0.56**	0.74	**0.81**	0.44	**0.46**	0.52	**0.73**
	TOX21_ERa_LUC_VM7_Agonist_10 nM_ICI182780	RF	DGCL	**0.61**	0.42	**0.97**	0.95	**0.7**	0.28	0.57	**0.86**
Estrogen-related receptor modulation	TOX21_PGC_ERR_Antagonist	XGB	DGCL	0.33	**0.39**	0.7	**0.76**	**0.33**	0.29	0.33	**0.66**
Extracellular matrix	BSK_3C_uPAR	LR	DGCL	0.5	**0.55**	0.62	**0.7**	0.41	**0.49**	0.63	**0.65**
	BSK_CASM3C_uPAR	LR	DGCL	0.32	**0.55**	0.63	**0.7**	0.33	**0.49**	0.32	**0.65**
	BSK_hDFCGF_TIMP1	RF	DGCL	0.34	**0.38**	0.64	**0.69**	**0.38**	0.26	0.33	**0.74**
	BSK_KF3CT_TIMP2	DT	DGCL	0.39	**0.42**	0.61	**0.72**	0.35	**0.37**	0.46	**0.58**
Other developmental signaling transcription factors	ATG_BRE_CIS	RF	DGCL	0.28	**0.34**	0.64	**0.72**	0.2	**0.23**	0.49	**0.66**
Oxidative stress	ATG_HIF1a_CIS	RF	DGCL	0.26	**0.35**	0.59	**0.71**	0.18	**0.23**	0.48	**0.77**
p53 signaling pathway	TOX21_p53_BLA_p2_ratio	LR	DGCL	0.26	**0.35**	0.6	**0.75**	0.22	**0.25**	0.32	**0.65**
Progesterone metabolic process	CEETOX_H295R_OHPROG	DT	MolFormer	0.65	**0.65**	0.63	**0.64**	0.51	**0.51**	**0.91**	0.9
	CEETOX_H295R_PROG	RF	DGCL	**0.55**	0.54	0.64	**0.69**	**0.53**	0.43	0.58	**0.8**
Progesterone receptor modulation	TOX21_PR_BLA_Antagonist_ratio	RF	DGCL	0.63	**0.68**	0.78	**0.82**	0.6	**0.61**	0.68	**0.77**
Retinoic acid receptor modulation	ATG_DR5_RAR_CIS	XGB	DGCL	0.2	**0.29**	0.59	**0.69**	0.16	**0.21**	0.32	**0.57**

Overall, the DL models achieved higher F1-score ranges (0.32–0.61), with the DGCL architecture most frequently selected as the top-performing model across different assay types. When compared with the best ML baseline for each assay, DGCL exhibited consistent performance gains, with F1-score improvements ranging from 0.05 to 0.30 depending on the endpoint. Notably, substantial gains were observed for extracellular matrix–related assays such as BSK_CASM3C_uPAR, where the DGCL model improved the F1-score by 0.23 relative to the best ML algorithm. In addition, the DL models achieved higher values for other evaluation metrics, including ROC-AUC, precision, and recall, indicating an overall enhancement in classification robustness. These findings suggest that the deep learning framework, through its graph-based representation learning, effectively captures structural and mechanistic features relevant to toxicity, resulting in superior predictive performance even under severe class imbalance (Cremer et al., 2023).

#### 3.2.2 Impact of multi-task learning on model performance

To assess whether learning shared representations across multiple endpoints could improve model performance, we compared the results of single-task and multi-task deep learning frameworks using the same ToxCast bioassay dataset. Since the DGCL architecture consistently showed the best performance across most assays (Table 3), it was adopted as the backbone model for this comparison. Overall, both configurations yielded similar average F1-scores indicating that multi-task learning did not markedly enhance mean performance (Supplementary Figure S2).

Minor improvements were observed for a few data-sparse assays, suggesting that shared representation learning may offer slight benefits when active class samples are limited (Supplementary Table S6). However, these gains were modest, and the multi-task framework served primarily as a supplementary evaluation rather than a major contributor to model performance in this study.

In addition, y-randomisation analysis was performed using the multi-task DGCL model to assess whether assay-level performance exceeded chance. While most assays showed clear separation from randomized-label performance, 5 assays did not fully satisfy the predefined robustness threshold (Supplementary Table S2). Nevertheless, these assays were retained in the multi-task learning framework because they are mechanistically linked to key DART pathways. Excluding them would reduce the mechanistic coverage of the model and limit its ability to capture biologically relevant signals across related endpoints.

### 3.3 External validation using ReProTect chemicals

To evaluate whether the model can be applied to established reference chemicals relevant to DART, we performed an external validation using the curated chemical set from the ECVAM ReProTect program (Pazos et al., 2010). This analysis evaluated the reliability of predictions for chemicals with well-characterized developmental and reproductive toxicity outcomes, beyond the internal test results. For the ReProTect reference chemicals, overall DART predictions were generated using a consensus-based approach, in which predictions from the 23 mechanistic endpoint–specific models were aggregated using majority voting to derive a single binary outcome for each chemical. This aggregation strategy was based on the assumption that chemicals triggering bioactivity across multiple DART-relevant mechanisms are more likely to induce adverse developmental or reproductive effects at the organism level. Prior to performance evaluation, AD assessment was performed for each of the 23 mechanistic endpoint–specific models, and predictions falling outside the AD were excluded from the analysis. As a result, one chemical that fell outside the applicability domain of all models was excluded, and external validation was conducted on the remaining 90 ReProTect chemicals.

**FIGURE 3:**
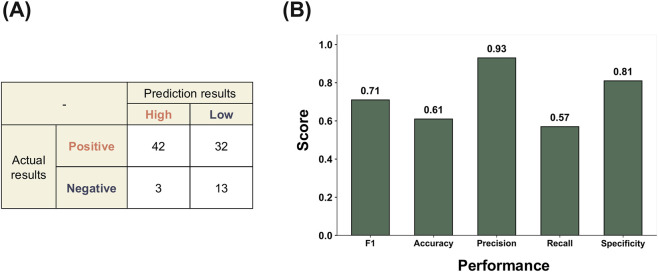
External validation performance of the multi-task deep learning model using ReProTect reference chemicals relevant to DART. **(A)** Confusion matrix summarizing consensus-based predictions, where overall DART outcomes were derived by majority voting across the 23 ToxCast DART-related bioassays. Only chemicals within the AD were included in the evaluation. **(B)** External performance metrics derived from the consensus predictions, with exact metric values shown above each bar. Full prediction results across all assays, including applicability domain classification, are provided in Supplementary Table S7.

As shown in Figure 3A, the confusion matrix illustrates that the model correctly identified a portion of both positive and negative chemicals, although misclassifications were still observed, particularly among the positive class. The external performance metrics showed that the model produced a comparable and balanced prediction profile across the evaluated indicators (Figure 3B). The F1-score remained at a level indicating that the trade-off between precision and recall was maintained in the external setting. Precision and specificity were relatively higher than the other metrics, showing that the model classified non-toxic chemicals conservatively while still detecting a portion of toxic chemicals.

Taken together, these external results showed F1-score performance comparable to the internal evaluation, while exhibiting a different precision–recall trade-off in the external validation context (Sharma et al., 2023). However, the modest recall observed for the external data indicates that sensitivity to some positive chemicals may still be limited, which should be considered when applying the model to broader chemical domains.

Collectively, the results of this study show the potential of AI models for mechanism-informed prediction of DART. The 23 assays used in this study were not randomly chosen for model training but were previously mapped to key mechanisms that are repeatedly implicated in DART, such as estrogen signaling and angiogenic responses. In current DART testing frameworks, these pathways are dispersed across multiple endpoints and species, which makes it difficult to detect them early with a single *in vivo* test (Burbank et al., 2025). By contrast, integrating these mechanism-linked assays enables the model to capture early cellular events associated with DART-related pathways, offering advantages for early-stage screening even though the model does not predict DART outcomes directly. Similar trends have also been observed in early-stage screening studies targeting endocrine-disrupting pathways such as AR and ER, where integrating high-throughput *in vitro* assays with computational models improved the identification of potential toxicants (Mansouri et al., 2016; Mansouri et al., 2020).

The DGCL-based framework effectively captured structural and mechanistic relationships within the ToxCast assays, yielding comparable or superior performance to conventional machine learning models. The application of multi-task learning further contributed to improving model stability, particularly for endpoints with limited active data. Similar improvements have also been reported for other toxicity endpoints, such as hepatotoxicity and cardiotoxicity, where deep learning models consistently outperformed conventional machine learning approaches in identifying compounds with adverse effects (Xu et al., 2015; Liu et al., 2025).

The external reference set enabled us to validate that the model performs reliably across chemicals with experimentally established DART outcomes. These validation results also provide practical insight into how such *in silico* tools should be interpreted. When tested with the reference chemicals from the ReProTect program, the model showed a balanced and relatively conservative prediction pattern, characterized by higher precision and lower recall compared to the internal evaluation. These features are appropriate for an early-stage screening tool meant to help prioritize chemicals rather than to make final negative decisions. Accordingly, the current prediction model is well positioned to serve as an initial component of a next-generation risk assessment (NGRA) pipeline, where it can be used to screen large chemical inventories, flag potential DART-related modes of action, and guide the selection of follow-up NAMs such as endocrine assays, embryoid body assays, or zebrafish embryo tests (Guidance Document for the Use of Adverse Outcome Pathways in Developing Integrated Approaches to Testing and Assessment, 2017; Kang et al., 2017; OECD, 2025; Bauer et al., 2021).

**FIGURE 4:**
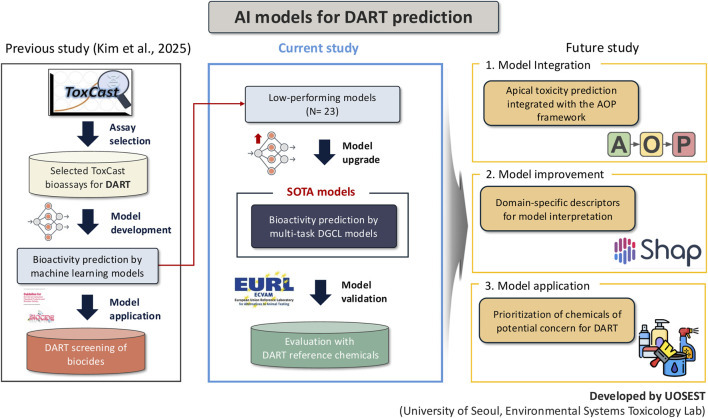
Conceptual framework of AI-based DART prediction.

Several limitations should be recognized. First, the dataset size and coverage were constrained by the availability of curated ToxCast assays mapped to DART-relevant mechanisms. This restriction may limit the diversity of biological processes represented and could reduce the model’s ability to capture rare toxicity mechanisms. Second, while the multi-task framework improved stability, the overall performance gains were modest, suggesting that shared representation learning alone may be insufficient to compensate for intrinsic data imbalance. Third, the external validation was conducted on a relatively small chemical set, and it remains uncertain how well the model would generalize to broader chemical domains with different structures, exposure routes, or metabolic profiles. Accordingly, the current study is positioned as a transitional step that upgrades previously low-performing DART-related bioactivity models to a state-of-the-art (SOTA) multi-task DGCL framework. By replacing conventional machine learning approaches with SOTA deep learning models optimized for multi-task bioactivity prediction, the present work establishes a robust foundation for further methodological advancement (Figure 4). Future efforts will build upon this upgraded framework by integrating class imbalance aware optimization strategies, hierarchical learning structures that reflect biological organization across assays, and additional biologically informative features such as toxicokinetic parameters or transcriptomic signatures (Kim et al., unpublished).

Nevertheless, this study provides a clear proof of concept for model upgrading and validation within a mechanism-informed DART prediction framework. By employing SOTA multi-task DGCL models, our approach demonstrates the ability to efficiently learn from complex and heterogeneous ToxCast assay data while preserving mechanistic relevance. Importantly, external validation using well-characterized DART reference chemicals confirms that the upgraded models achieve conservative predictive behavior suitable for early-stage screening. As larger and more comprehensively annotated DART-relevant assay datasets become available, and as AOP frameworks continue to expand and mature, this modeling strategy is expected to become increasingly interpretable through domain-specific descriptors and explainability tools, such as SHAP-based interpretation (Kim et al., unpublished). Ultimately, the integration of apical toxicity prediction within the AOP framework, together with validated bioactivity models, will enhance the applicability of *in silico* approaches for chemical prioritization in NGRA and NAM-based decision-making, thereby supporting reduced reliance on traditional animal testing without compromising protective intent.

## 4 Conclusion

This study presents a mechanism informed multi-task deep learning framework for predicting DART using curated ToxCast bioassays. By applying advanced deep learning architecture, the model demonstrated performance that exceeded traditional machine learning approaches and showed greater stability under severe data imbalance.

External validation using the ECVAM ReProTect reference chemicals showed robust and conservative predictive behavior, supporting the reliability of the model as a screening level tool within non animal safety assessment strategies. These results highlight the potential of combining mechanistic bioassay data and deep learning to advance mechanism based, human relevant approaches for DART evaluation, ultimately contributing to the reduction of animal testing in regulatory toxicology.

## Data Availability

All data are available as supporting information.
