# Supplementary material for: Multi-task deep learning models for mechanism-based prediction of developmental and reproductive toxicity (DART) using ToxCast bioassays
Source: Front Toxicol. 2026 Feb 4;8:1751644. doi: 10.3389/ftox.2026.1751644 (PMC12912713; doi:10.3389/ftox.2026.1751644)
Supplement: Supplementary file 2 [file Table1.docx]

**Supplementary information**

**Multi-Task Deep Learning Models for Mechanism-Based Prediction of Developmental and Reproductive Toxicity (DART) using ToxCast Bioassays**

*Siyeol Ahn^1^, Hojun Jung^3^, Jinwon Hwang^1^, Donghyeon Kim^1^, Hyunjun Kim^3^, Wooseok Kim^3^, Yunjung Lee^3^, Changwon Lim^2,3^*, Jinhee Choi^1^**

^1^School of Environmental Engineering, University of Seoul, 163 Seoulsiripdae-ro, Dongdaemun-gu, Seoul 02504, Republic of Korea

^2^ Department of Smart Cities, Chung-Ang University, 84 Heukseok-ro, Dongjak-gu, Seoul 06974, Republic of Korea

^3^ Department of Statistics and Data Science, Chung-Ang University, 84 Heukseok-ro, Dongjak-gu, Seoul 06974, Republic of Korea

**Summary:** 11 pages, 2 figures.

**Supplementary Information 1.** Mathematical formulations of Section 2.3 (Molecular representation learning).

**Figure S1.** Multi-task training and validation loss curves for the DGCL model.

**Figure S2.** Impact of multi-task learning on model performance using the DGCL backbone.

**Supplementary Information 1.** Mathematical formulations of Section 2.3 (Molecular representation learning)

This supplementary Information summarizes the mathematical formulations of the molecular representation learning models, including generic message passing framework – MPNN, GCN, GIT, GAT – and the four models applied in this study – TransFoxMol, DGCL, MolPath, Molformer. A molecule is represented as a graph $G=\left( V, E \right)$ where V represents node (atom) and E represents edge (bond).

**(1) Message Passing Neural Network**

In Message Passing Neural Network (MPNN) formulation, each node $v\in V$updates its hidden state $h_{v}^{(t)}$ by aggregating information from its neighboring atoms $u\in N\left( v \right)$ (Gilmer et al., 2017). This process is formulated as:

$$m_{v}^{t+1}=\sum_{w\in N\left( v \right)} M_{t}\left( h_{v}^{t},h_{w}^{t},e_{vw} \right)$$

$h_{v}^{t+1}=U_{t}\left( h_{v}^{t},m_{v}^{t+1} \right)$,

where $e_{vw}$ is an edge feature, and $M_{t}$ , and $U_{t}$ denote learnable message and update functions. After $t$ iterations, a readout function $R(\cdot)$ aggregates all node embeddings into a global molecular representation:

$$\hat{y}=R\left( \left\{ h_{v}^{T} |v\in G \right\} \right),$$

which encodes the molecule’s overall structural and chemical characteristics.

**(2) Graph Convolutional Network**

Graph Convolutional Network (GCN) (Kipf and Welling, 2016) is a specific instance of MPNN that implements neighborhood aggregation as a spectral graph convolution based on the normalized Laplacian:

$$H^{(l+1)}=\sigma\left( \tilde{D}^{-\frac{1}{2}}\tilde{A} \tilde{D}^{-\frac{1}{2}}H^{\left( l \right)}W^{\left( l \right)} \right),$$

which effectively diffuses atomic features over immediate neighbors in the molecular graph.

Here, $H^{(l)}\in\mathbb{R}^{N\times D}$denotes the matrix of atom embeddings at layer $l$, where each row corresponds to an atom and $d_{l}$ is the hidden dimension. The matrix $\tilde{A}=A+I$ is the adjacency matrix of the graph with added self-connections, and $\tilde{D}$ is its diagonal degree matrix with entries $\tilde{D}_{ii}=\sum_{j} \tilde{A}_{ij}$. The term $\tilde{D}^{-\frac{1}{2}}\tilde{A}\tilde{D}^{-\frac{1}{2}}$ represents the symmetrically normalized Laplacian, which determines how information is scaled and propagated between neighboring atoms*.* $W^{(l)}$ is a learnable weight matrix, and $\sigma(\cdot)$ is nonlinear activation function*.*

**(3) Graph Isomorphism Network**

Graph Isomorphism Network (GIN) enhances the expressive power of GCN by using an injection aggregation scheme (Xu et al., 2018). $k$th layer updates the representations of an atom $v$ as:

$$h_{v}^{\left( k \right)}=MLP^{\left( k \right)}\left( \left( \left( 1+\epsilon^{\left( k \right)} \right)\cdot h_{v}^{\left( k-1 \right)}+\sum_{u\in N\left( v \right)} h_{u}^{\left( k-1 \right)} \right) \right),$$

where $h_{v}^{(k-1)}$denotes the hidden representation of atom $v$ at layer $k-1$, $\mathcal{N(}v)$ is the set of neighboring atoms, $\epsilon^{(k)}$is a learnable parameter controlling the relative weight of the central atom versus its neighbors, and $\mathrm{MLP}^{(k)}(\cdot)$is a multilayer perceptron applied after sum aggregation. This mechanism ensures GIN to distinguish subtle topological variations such as branching, geochemical, configurations and isomeric topology. It makes GIN effective in molecular property prediction tasks requiring high-resolution substructure discrimination.

**(4) Graph Attention Network**

Graph Attention Network (GAT) incorporates attention weights to modulate the relative influence of neighboring atoms (Veličković et al., 2017). For a central atom $i$ and one of its neighbors $j$, the normalized attention coefficient is computed as:

$$\alpha_{ij}=\frac{exp \left( LeakyReLU\left( \vec{a^{T}}\left[ W\vec{h_{i}}|W\vec{h_{j}} \right] \right) \right)}{\sum_{k\in N_{i}} exp \left( LeakyReLU\left( \vec{a^{T}}\left[ W\vec{h_{i}}|W\vec{h_{k}} \right] \right) \right)},$$

where $\mathbf{h}_{i}$ and $\mathbf{h}_{j}$are the input feature vectors of atoms $i$ and $j$, $W$ is a learnable linear projection, $\mathbf{a}$ is a learnable attention vector, $[\cdot\text{|}\cdot]$ denotes vector concatenation, and $\mathcal{N}_{i}$is the 1-hop neighborhood of $i$. This enhances its ability to prioritize chemically salient interactions. Multi-head attention further allows the model to jointly consider multiple chemical perspectives thereby focusing the representation on structural elements most relevant to the molecular property prediction task.

**(5) TransFoxMol**

Transfoxmol integrates graph convolution layer with a transformer encoder to jointly encode local and global structure (Gao et al., 2023). Its local embedding is computed using a graph convolution layer modulated by a local interaction map $M_{j,i}$:

$$M_{j,i}=\left\{ \begin{aligned} Adj_{j,i}Adj_{j,i}> 0 \\ \frac{1}{dist_{w}^{Dist_{j,i}}}& Adj_{j,i}= 0 \end{aligned} \right.,$$

where $\mathrm{Adj}_{j,i}$denotes the element of the interaction map $\mathrm{Adj}\in\mathbb{R}^{N_{\text{atoms}}\times N_{\text{atoms}}}$encoding bonded or conjugated interactions between atoms $j$and $i$, $\mathrm{Dist}_{j,i}$is the shortest path distance between atoms $j$and $i$on the 2D molecular graph, and $\mathrm{dist}_{w}$is a distance-attenuation constant that controls how rapidly long-range interactions decay. Thus, directly bonded or conjugated pairs retain their chemically meaningful weights via $\mathrm{Adj}_{j,i}$, whereas non-bonded pairs are down-weighted according to their topological distance, allowing TransFoxMol to encode both local bonding patterns and attenuated long-range interactions in a unified matrix $M$. Local atomic embeddings are computed through a graph convolution layer modulated by the local map $\mathrm{Loc}$, which stores bond-type–specific edge weights. This process is mathematically defined as:

$$h_{i}^{\left( l \right)}=W_{1}^{\left( l \right)}h_{i}^{\left( l-1 \right)}+W_{2}^{\left( l \right)}\sum_{j\in N\left( i \right)} \text{Loc}_{c_{j,i}}\cdot h_{j}^{\left( l-1 \right)},$$

where $h_{i}^{(l-1)}$and $h_{j}^{(l-1)}\in\mathbb{R}^{d_{\text{hidden}}}$are the features of atoms $i$and $j$from the $(l-1)$th layer, $h_{i}^{(l)}$is the updated embedding of atom $i$at layer $l$, $\mathcal{N}\left( i \right)$ denotes the set of neighboring atoms of $i$, and $W_{1}^{(l)},W_{2}^{(l)}\in\mathbb{R}^{d_{\text{hidden}}\times d_{\text{hidden}}}$are trainable projection matrices. ${Loc}_{j,i}$ are edge weights assigned based on bond type. These atom-level embeddings are then passed to a Focused Dot Product Attention layer that incorporates the global map M into the transformer encoder:

$$\text{Attention}\left( Q,K,V \right)=\text{softmax}\left( \frac{QK^{\top}}{\surd d}\cdot M \right)V.$$

Here, $Q,K,V\in\mathbb{R}^{N_{\text{atoms}}\times d}$ are the query, key, and value matrices obtained by linear projections of the atom embeddings, $d$ is the hidden dimensionality, and $M\in\mathbb{R}^{N_{\text{atoms}}\times N_{\text{atoms}}}$ is the global map defined above. This mechanism strengthens attention between atom pairs with strong structural interactions, enabling TransFoxMol to generate molecular embeddings that unite local chemical environments with long-range dependencies relevant to DART mechanisms. The final readout operation aggregates these features into a molecule-level embedding, which is then used as the input to the downstream MLP classifier for DART assay prediction.

**(6) DGCL**

DGCL is a self-supervised learning framework based on contrastive learning (Jiang et al., 2024). Two different GNN encoders - GIN and GAT - process the same molecular graph generating two distinct latent vectors $h_{GIN}^{i}$ and $h_{GAT}^{i}$. Both representations are then projected onto $z_{GIN}^{i}$ and $z_{GAT}^{i}$ through a nonlinear projection head and the similarity $sim\left( z_{GIN}^{i}, z_{GAT}^{i} \right)$ between two projections is then determined. To enforce agreement between two encoders while distinguishing different molecules within the batch, DGCL optimizes the contrastive loss, which is defined as:

$$\mathcal{L=-}\log\left( \frac{E\left( z_{\text{GIN}}^{i},z_{\text{GAT}}^{i} \right)}{\sum_{k=1}^{N} \mathbb{1}_{\left[ k\neq i \right]}E\left( z_{\text{GIN}}^{i},z_{\text{GIN}}^{k} \right)+\sum_{k=1}^{N} E\left( z_{\text{GIN}}^{i},z_{\text{GAT}}^{k} \right)} \right),$$

$$E\left( X,Y \right)= exp\left( \frac{sim\left( X,Y \right)}{\tau} \right).$$

This method pulls together the representations of the same molecule to form a positive pair, while pushing away representations belonging to different molecules which constitute negative pairs. Through this dual-encoder contrastive mechanism, DGCL learns invariant and structurally robust molecular embeddings before being finetuned to the supervised DART prediction task. For the downstream DART task, the graph-level embeddings from the GIN and GAT encoders are concatenated with molecular fingerprints to construct a unified feature vector, which is used as the input to the downstream MLP classifier.

**(7) MolPath**

MolPath is a supervised, chain aware GNN that learns molecular representations by propagating information along shortest paths (12). For each molecule, MolPath enumerates shortest paths of length k, $\delta^{(k)}=\{\delta_{1}^{\left( k \right)}, \delta_{2}^{\left( k \right)}, \ldots, \delta_{m_{k}}^{\left( k \right)} \}$ and applies Initial Residual Difference Connection (IRDC) to focus on newly gained information. IRDC is formulated as:

$$\tilde{H}_{\delta_{i}^{\left( k \right)}} =IRDC^{\left( k \right)}\left( H_{\delta_{i}^{\left( k \right)}}^{\left( 0 \right)},\sum_{j=1}^{k-1} H_{\delta_{i}^{\left( k \right)}}^{\left( j \right)} \right)$$

$$=\left( 1-\lambda\right)H_{\delta_{i}^{\left( k \right)}}^{\left( 0 \right)}-\lambda\sum_{j=1}^{k-1} H_{\delta_{i}^{\left( k \right)}}^{\left( j \right)},$$

where $H_{\delta_{i}^{\left( k \right)}}^{\left( 0 \right)}$ is the initial node feature sequence for path $\delta_{i}^{\left( k \right)}$, and $H_{\delta_{i}^{\left( k \right)}}^{\left( j \right)}$ is the feature of path’s nodes after the $j$th convolutional layer. IRDC refined sequence is then applied into an LSTM to capture ordered, chain-like dependencies among atoms:

$H_{\delta_{i}^{\left( k \right)}}=LSTM\left( \tilde{H}_{\delta_{i}^{\left( k \right)}}, \delta_{i}^{\left( k \right)} \right),$

and these path-level features are pooled and aggregated to update node embeddings at layer k:

$H^{\left( k \right)}= \phi(NORM\left( H^{\left( k-1 \right)}+ \sum_{j=1}^{k-1} H_{\delta_{i}^{\left( k \right)}}^{\left( j \right)} \right).$

To reflect that different path lengths contribute unevenly to the learned representation, Molpath applies a path-attention mechanism over shortest paths of different orders. Using the initial node embedding $H^{(0)}$ as the query, the attention weight for order $i$ is computed as:

$$\omega_{i}=\text{softmax}\left( \frac{\left( W_{\text{Att}}\cdot H^{\left( 0 \right)} \right)^{\top}\cdot\left( W_{\text{Att}}\cdot H^{\left( i \right)} \right)}{\sqrt{d}} \right),$$

and the final graph-level molecular representation is obtained by pooling over all nodes after combining the initial features with the attention-weighted shortest-path features:

$$H_{g}=Pool\left( H^{\left( 0 \right)}+ \sum_{i=1}^{k} \omega_{i}H^{\left( i \right)} \right).$$

In this way, MolPath learns molecular representations that emphasize informative chain-like and long-range structural patterns frequently implicated in toxicity. The resulting molecule-level representation $H_{g}$ is then used as the input to the downstream MLP classifier for DART assay prediction.

**(8) ChemRL-GEM**

ChemRL-GEM is a representative example of geometry-based molecular representation learning (Fang et al., 2022). In this model, a molecule is represented as a 3D molecular graph $G=\left( V, E, P \right),$ where $V$ and $E$ denote atoms and bonds as in 2D graphs, and $P=\{\mathbf{P}_{u}\in\mathbb{R}^{3}\mid u\in V\}$ stores atomic coordinates. To incorporate this geometric information, the message function is typically modified so that each atom aggregates both chemical features and 3D geometric descriptors. It employs a geometry-enhanced GNN (GeoGNN) which constructs an atom-bond graph and a bond-angle graph, allowing bond representations to absorb local geometric information: bond lengths and bond angles. At the atom-bond level, the representation of an atom $u$ at layer $k$ is updated by combining its previous state $h_{u}^{(k-1)}$ with an aggregated message $a_{u}^{(k)}$ that already encodes geometry-enriched bond feature:

$$h_{u}^{(k)}=COMBINE_{atom-bond}^{\left( k \right)}\left( h_{u}^{\left( k-1 \right)}, a_{u}^{\left( k \right)} \right).$$

After $K$ iterations of such geometry-aware message passing, a permutation invariant $READOUT$ function pools the final atom embeddings into a graph-level molecular vector:

$$h_{G}=READOUT\left( h_{u}^{\left( K \right)} \mid u\in V \right),$$

which serves as a compact geometry-informed representation of the molecule. In other words, bond and angle information are progressively propagated into atom states through $a_{u}^{(k)}$, and the $READOUT$ operation integrates these geometry-enhanced atom features into a single molecular embedding.

**(9) Molformer**

Molformer is a geometry-based model that directly integrates 3D coordinates into a transformer-based architecture on heterogeneous molecular graphs (Wu et al., 2023). A molecule is encoded as $S=\left( P, H \right),$ where $P=\{ p_{i}{\}}_{i=1}^{N}\in R^{N \times3}$denotes atom or motif coordinates and $H=\{ h_{i}{\}}_{i=1}^{N}$ indicate roto-translationally invariant features, which are projected to initial node embeddings $v_{i}$. From $P,$ Molformer computes the Euclidean distances $d_{i,j}= \parallel P_{i}-P_{j}\parallel_{2}$ and applies a shallow convolution to obtain a distance feature $\hat{d}_{ij}.$In the heterogeneous self-attention layer, the attention logit between nodes $i$ and $j$ is then modulated by this geometry term:

$$\hat{a}_{i,j}=\left( \frac{q_{i}k_{j}^{T}}{\sqrt{\psi_{model}}} \right)\cdot\hat{d}_{i,j}+b_{\emptyset(i.j)},$$

where $\mathbf{q}_{i}$, $\mathbf{k}_{j}$and $\mathbf{v}_{j}$are query, key and value vectors obtained from $\mathbf{v}_{i}$, and $b_{\phi(i,j)}$is a type-dependent bias for atom–atom, atom–motif or motif–motif pairs. To capture local 3D context at different spatial ranges, Molformer further introduces distance thresholds $\{\tau_{s}\}$ and defines scale-specific attention and representation as:

$$a_{ij}^{\tau_{s}}= \hat{a}_{ij}\cdot1_{\{d_{i,j} < \tau_{s}\}}, z_{i}^{\tau_{s}}= \sum_{j=1}^{N} \sigma\left( a_{ij}^{\tau_{s}} \right) v_{j},$$

where $1_{\{d_{i,j} < \tau_{s}\}}$ is the indicator function. The set of local multi-scale features $\{\mathbf{z}_{i}^{\tau_{s}}{\}}_{s}$is concatenated with a global attention output and passed through a feed-forward network to yield refined geometry-aware node embeddings. Finally, an Attentive Farthest Point Sampling (AFPS) readout selects a subset of spatially diverse and attention-important nodes and aggregates their refined embeddings $\{\boldsymbol{z}_{i}^{''}{\}}_{i\in\mathcal{P}}$ via global pooling to obtain the final molecular vector. Through this combination of distance-modulated attention, multi-scale geometric masking and AFPS-based readout, Molformer learns 3D-aware molecular representations that tightly integrates spatial organization with atom and motif-level chemistry, which are subsequently used for DART-related toxicity prediction.

**
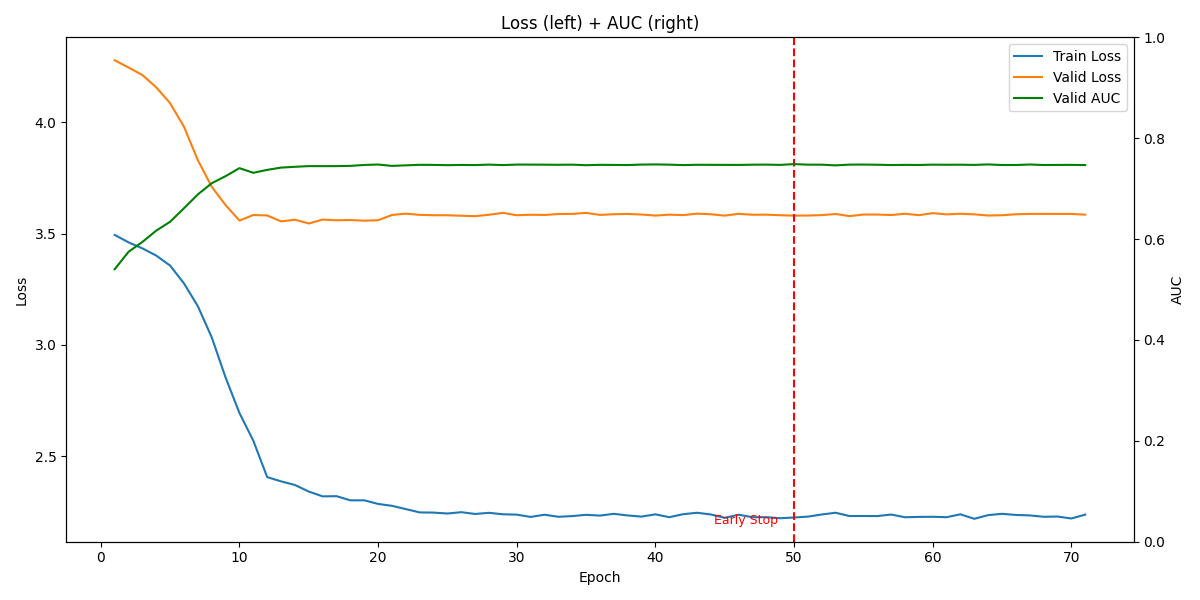
**

**Figure S1.** Multi-task training and validation loss curves for the DGCL model. The validation loss decreased rapidly during early epochs and stabilized around epoch 14.

**
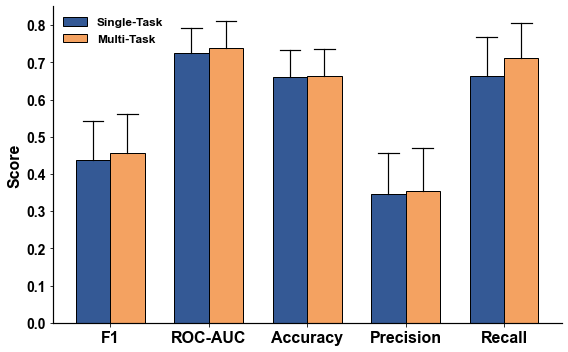
**

**Figure S2.** Impact of multi-task learning on model performance using the DGCL backbone. Bars represent the average performance across individual assays, and error bars indicate standard deviations.

Fang, X., Liu, L., Lei, J., He, D., Zhang, S., Zhou, J., et al. (2022). ChemRL-GEM: Geometry Enhanced Molecular Representation Learning for Property Prediction. *Nat Mach Intell* 4, 127–134. doi: 10.1038/s42256-021-00438-4

Gao, J., Shen, Z., Xie, Y., Lu, J., Lu, Y., Chen, S., et al. (2023). TransFoxMol: predicting molecular property with focused attention. *Brief Bioinform* 24. doi: 10.1093/BIB/BBAD306

Gilmer, J., Schoenholz, S. S., Riley, P. F., Vinyals, O., and Dahl, G. E. (2017). Neural Message Passing for Quantum Chemistry. *34th International Conference on Machine Learning, ICML 2017* 3, 2053–2070. Available at: https://arxiv.org/pdf/1704.01212 (Accessed November 18, 2025).

Jiang, X., Tan, L., and Zou, Q. (2024). DGCL: dual-graph neural networks contrastive learning for molecular property prediction. *Brief Bioinform* 25. doi: 10.1093/BIB/BBAE474

Kipf, T. N., and Welling, M. (2016). Semi-Supervised Classification with Graph Convolutional Networks. *5th International Conference on Learning Representations, ICLR 2017 - Conference Track Proceedings*. Available at: https://arxiv.org/pdf/1609.02907 (Accessed November 18, 2025).

Veličković, P., Casanova, A., Liò, P., Cucurull, G., Romero, A., and Bengio, Y. (2017). Graph Attention Networks. *6th International Conference on Learning Representations, ICLR 2018 - Conference Track Proceedings*. doi: 10.1007/978-3-031-01587-8_7

Wu, F., Radev, D., and Li, S. Z. (2023). Molformer: Motif-Based Transformer on 3D Heterogeneous Molecular Graphs. *Proceedings of the AAAI Conference on Artificial Intelligence* 37, 5312–5320. doi: 10.1609/AAAI.V37I4.25662

Xu, K., Jegelka, S., Hu, W., and Leskovec, J. (2018). How Powerful are Graph Neural Networks? *7th International Conference on Learning Representations, ICLR 2019*. Available at: https://arxiv.org/pdf/1810.00826 (Accessed November 18, 2025).
